# Associations between breastfeeding and the “10 Steps for Successful Breastfeeding”

**DOI:** 10.1093/eurpub/ckac131.430

**Published:** 2022-10-25

**Authors:** M Economou, O Kolokotoni, E Hadjigeorgiou, I Paphiti-Demetriou, V Hadjiona, C Kouta, E Lambrinou, I Hadjipanteli, N Middleton

**Affiliations:** 1 Department of Nursing, Cyprus University of Technology, Limassol, Cyprus; 2 Cyprus Breastfeeding Association “Gift for Life”, Nicosia, Cyprus; 3 Archbishop Makarios Pediatric Hospital, Nicosia, Cyprus

## Abstract

**Background:**

The Baby-Friendly Hospital Initiative’s (BFHI) ‘‘Ten Steps for Successful Breastfeeding'’ has been the cornerstone of national and international strategies for decades; however, adherence is suboptimal. Despite successive National Strategies, breastfeeding rates remain low in Cyprus.

**Methods:**

The overall experience of a consecutive sample of 568 mother-baby dyads (response: 70.8%) across all public (N = 5) and 29 (of 35) private maternity clinics was operationalized as the sum score of full, partial or no implementation of each WHO/UNICEF BFHI self-assessment questionnaire item, with the exclusion of Step 6 (exclusivity). Associations with initiation and continuation of any (BF) and exclusive breastfeeding (EBF) up to the 6th month were explored in logistic regression after adjusting for potential confounders, including breastfeeding self-efficacy.

**Results:**

At mean score 5.6 (SD = 2.4), the overall 10 steps experience was low (theoretical range 0-14), even among those who breastfed exclusively (M = 6.9, SD = 2.1; p < 0.001). EBF and BF initiation and continuation showed a stepwise association with self-reported experience of the 10 steps. Across quartiles of increasing scores, the prevalence of EBF was 7.1%, 15.1%, 17.0% and 35.6%. The quartile of mothers who assessed their experience more favourably were 8- (adjOR: 8.4, 95% CI 1.5-48.0; p = 0.017) and 4-times (adjOR: 4.1; 95% CI 1.7-9.8, p = 0.002) more likely to initiate BF and EBF, even though only 6.5% continued EBF by the 6th month. Step 7 (rooming-in) and step 9 (no pacifiers) were least practiced but more strongly associated with EBF initiation.

**Conclusions:**

While breastfeed intention may determine the actual experience of the 10 steps, implementation across maternity clinics appeared fragmented, despite clear association with successful initiation and continuation of BF, at the same time that the Cyprus National Committee for Breastfeeding is embarking on the first BFHI accreditation of maternity clinics.

**Key messages:**

• Despite successive National Strategies identifying breastfeeding as Public Health priority, adherence to the WHO/UNICEF’s 10 Steps appears fragmented across Cypriot maternity clinics.

• Despite low overall adherence, mothers reporting experiencing more of the 10 steps were more likely to initiate and continue breastfeeding.

